# Understanding Capabilities, Opportunities, and Motivations of Walking for Physical Activity Among Adults With Intellectual Disabilities: A Qualitative Theory‐Based Study

**DOI:** 10.1111/jar.70105

**Published:** 2025-07-30

**Authors:** Sophie C. Westrop, Ailsa Niven, Craig Melville, Donna‐Marie Speir, Arlene M. McGarty

**Affiliations:** ^1^ School of Education, Language and Psychology York St John University York UK; ^2^ Mental Health and Wellbeing University of Glasgow Glasgow Scotland; ^3^ Moray House of Education and Sport University of Edinburgh Edinburgh Scotland; ^4^ Values Into Action Scotland Glasgow Scotland

## Abstract

**Background:**

This study aimed to apply the COM‐B model to understand the **c**apabilities, **o**pportunities, and **m**otivations for walking **b**ehaviour among adults with intellectual disabilities.

**Methods:**

A qualitative study was conducted with adults (≥ 18 years) with mild to moderate intellectual disabilities living in Greater Glasgow using one‐to‐one interviews (*n* = 12; women = 5) and a photo‐elicitation activity followed by a focus group discussion (*n* = 5; women = 1). The framework approach to analysis allowed for the influences of walking to be mapped onto the COM‐B model.

**Results:**

Walking is a complex behaviour with many capabilities, opportunities and motivations to consider. Adults with intellectual disabilities were involved in making decisions about what results should be prioritised.

**Conclusions:**

The COM‐B model is a flexible framework that can be applied to understand health behaviours of adults with intellectual disabilities. It is imperative to work with adults with intellectual disabilities throughout the research process.

**Summary**

- The COM‐B model is a theoretical framework used to understand capabilities, opportunities, and motivations for a behaviour.
- This study used interviews, photo‐based methods, and a focus group with adults with learning disabilities to apply the COM‐B to walking for physical activity.
- There are many different capabilities, opportunities, and sources of motivation identified that impact on walking for adults with learning disabilities.
- From the perspective of adults with learning disabilities, the important findings were that walking is better when walking in a group, walking should have a purpose (e.g., walking to the shops), that everyone has individual needs, and everyone needs to feel included.

## 1 Introduction

Walking is a free form of physical activity associated with reduced risk for all‐cause mortality, obesity, diabetes, and major depressive disorder (Kelly et al. 2018; Master et al. 2022; Paluch et al. 2022). Accessing the outdoors when walking may also indirectly improve wellbeing, as accessing green and blue spaces has been linked to improved mental health (Geary et al. 2023). Subsequently, walking for physical activity is an important behaviour to consider when trying to alleviate the risk of health inequalities experienced by adults with intellectual disabilities, which include reduced life expectancy, poor general health, and mental ill health (O'Leary et al. 2018; Hughes‐McCormack et al. 2018).

Previous interventions to increase walking and physical activity of adults with intellectual disabilities have reported limited effectiveness (Melville et al. 2015; Rana et al. 2024). The physical activity levels of adults with intellectual disabilities are very low, with many multi‐level barriers impacting on participation (Bossink et al. 2017; Dairo et al. 2016; Westrop et al. 2019). For example, having less autonomy to choose walking routes and a reliance on social support (Mitchell et al. 2018). The importance of social support and reduced autonomy is attributed to the limitations in intellectual functioning and adaptive skills needed for living independently, which are experienced by adults with intellectual disabilities (American Association of Intellectual and Developmental Disabilities, AAIDD 2021).

Testable theoretical frameworks can help to understand how to improve physical activity and walking. The most frequently used theories in lifestyle change interventions with adults with intellectual disabilities are the Transtheoretical Model, Social Cognitive Theory, and Theory of Planned Behaviour (Rana et al. 2024). However, these theoretical models do not incorporate all potentially relevant influences of behaviour (Michie et al. 2014). The greater focus on internal processes, such as motivational constructs and perceived ability, has little consideration of the nuanced barriers to behaviour change experienced by adults with intellectual disabilities (Maenhout et al. 2024; Pitchford et al. 2018; Rana et al. 2024; Westrop et al. 2024). For example, autonomy and freedom of choice, barriers to social support, and factors relating to adaptive behaviours, such as practical, conceptual, and social skills (Westrop et al. 2024).

In recent years, researchers have been using a specific theoretical framework called the COM‐B model to understand the social support caregivers provide for physical activity for adults with intellectual disabilities (Bossink et al. 2019, 2020; Overwijk et al. 2021), the physical activity of adolescents with intellectual disabilities (Maenhout et al. 2024; McDermott et al. 2022; Mulhall et al. 2024), and the evaluation of a fitness programme for adults with intellectual disabilities (Savage and Colombo‐Dougovito 2023). The COM‐B model can be adapted to specific populations and behaviours and postulates that interacting **c**apabilities, **o**pportunities, and **m**otivations influence a **b**ehaviour (Table 1; Michie et al. 2014).

**TABLE 1:** Summary of COM‐B model.

COM‐B component		Definition
Capabilities	Physical	Strength; stamina; physical skills
	Psychological	Knowledge or psychological skills; mental processes
Opportunities	Physical	Environment, e.g., time, resources, etc.
	Social	Interpersonal influences, cultural norms, etc.
Motivation	Automatic	Automatic processes, such as emotional reactions
	Reflective	Reflective processes, such as making plans and evaluations

*Note:* COM‐B model components (Michie et al. 2014).

At the centre of a wider framework called the Behaviour Change Wheel, which is used to guide intervention development (BCW; Michie et al. 2014), the COM‐B enables a behavioural diagnosis to be developed to understand a behaviour and inform intervention design (Michie et al. 2014). Limited existing physical activity and walking interventions for adults with intellectual disabilities have been informed by theory, and there has been no previous application of the COM‐B to develop a behavioural diagnosis of walking. Therefore, this study aims to address this gap in the literature by addressing the following research question: What are the capabilities, opportunities, and motivations experienced by adults with mild to moderate intellectual disabilities for walking?

## 2 Methods

### 2.1 Ethical Approval and Ethical Considerations

Ethical approval was provided by the University of Glasgow College of Medical, Veterinary, and Life Sciences ethics committee. Additional procedures were in place to facilitate informed consent, such as producing *all* materials in an Easy Read format, providing additional time to read materials and to ask questions, and ensuring participants understood what the study involved. Participants were also able to invite a trusted adult source of support to join them in the interviews.

### 2.2 Research Team

All research team members have expertise in working with adults with intellectual disabilities and/or lifestyle modification. The team included a representative from the non‐profit organisation Values into Action Scotland (VIAS) which supports adults with intellectual disabilities to achieve their goals. Involvement of VIAS helped to ensure the research was relevant and accessible for people with intellectual disabilities.

The researcher primarily responsible for data collection and analysis aligns with the philosophical stance of critical realism. Critical realism argues that reality exists independent of the researcher and understanding is shaped by human experiences; however, this understanding can be improved by rigorous research (Lyons and Coyle 2021). The researcher's gender identity of being a female/woman may have shaped the research as women involved in the study may have been more comfortable sharing personal experiences of walking.

### 2.3 Design

The qualitative study design enabled understanding of the capabilities, opportunities and motivations for walking from the perspective of adults with intellectual disabilities. As the COM‐B is used in the context of behaviour change interventions, the use of qualitative methods ensured the influences of walking identified reflected people's lived experiences (O'Cathain et al. 2019; Westrop et al. 2024).

The study included two data collection pathways, both adopting photo‐elicitation methods. Pathway one: semi‐structured interviews (in‐person or using remote methods) were conducted with researcher‐directed photo‐elicitation methods. Pathway two: participant‐directed photo‐elicitation methods followed by a focus group to discuss the photographs taken. Throughout the process, a reflexive approach to data collection and analysis ensured the process was inclusive and accessible.

### 2.4 Sampling and Recruitment

Purposive sampling sought adults (≥ 18 years) who identified as having mild to moderate intellectual disabilities and lived in the Greater Glasgow area. Recruitment and data collection took place between August 2022 and May 2023. Based on the recommendation of VIAS, a small financial incentive of a £20 gift voucher was used.

For pathway one, a provisional sample of *n* = 12 (50% women) was sought for the interviews based on sampling recommendations for identifying themes in qualitative research (Guest et al. 2020). However, the final sample size was flexible and decided in situ based on the richness of the data (Braun and Clarke 2021). Prospective participants were recruited through VIAS and linked groups for adults with intellectual disabilities in Greater Glasgow.

For pathway two, the tentative sample was *n* = 6 adults with intellectual disabilities. This number has been considered appropriate for group discussions (Nyumba et al. 2018) and was appraised as feasible when including the presence of sources of support and the photo‐elicitation methodology. Pathway two involved collaborating with an existing walking group for people with intellectual disabilities. It is important to emphasise that the recruitment through a walking group was only in pathway two.

### 2.5 Data Collection

#### 2.5.1 Demographic Information

A demographic questionnaire collected data on participant age, gender identity, residential setting, and post code to calculate the Scottish Index of Multiple Deprivation (SIMD; Scottish Government 2020). Due to the recruitment strategy through Glasgow‐based groups for people with intellectual disabilities, the demographic questionnaire was brief to reduce the risk of participants being identifiable. The questionnaire was in an easy‐read format to allow completion by either the participant or researcher.

#### 2.5.2 Pathway One: Semi‐Structured Interviews With Researcher Directed Photo‐Elicitation

The interviews involved an initial photo‐elicitation activity and then followed a semi‐structured interview schedule reflecting the COM‐B behavioural diagnosis (see SI File 1; Michie et al. 2014). Photographs curated by the researcher were used to help elicit memories and shared understanding relating to the participants' experiences of walking (Glaw et al. 2017). The use of visual and photo‐based methods with people with intellectual disabilities can improve the accessibility of interviews for people with more complex communication needs and add context to what is discussed during the interview (Kenny et al. 2023; Sigstad and Garrels 2021).

**FIGURE 1:**
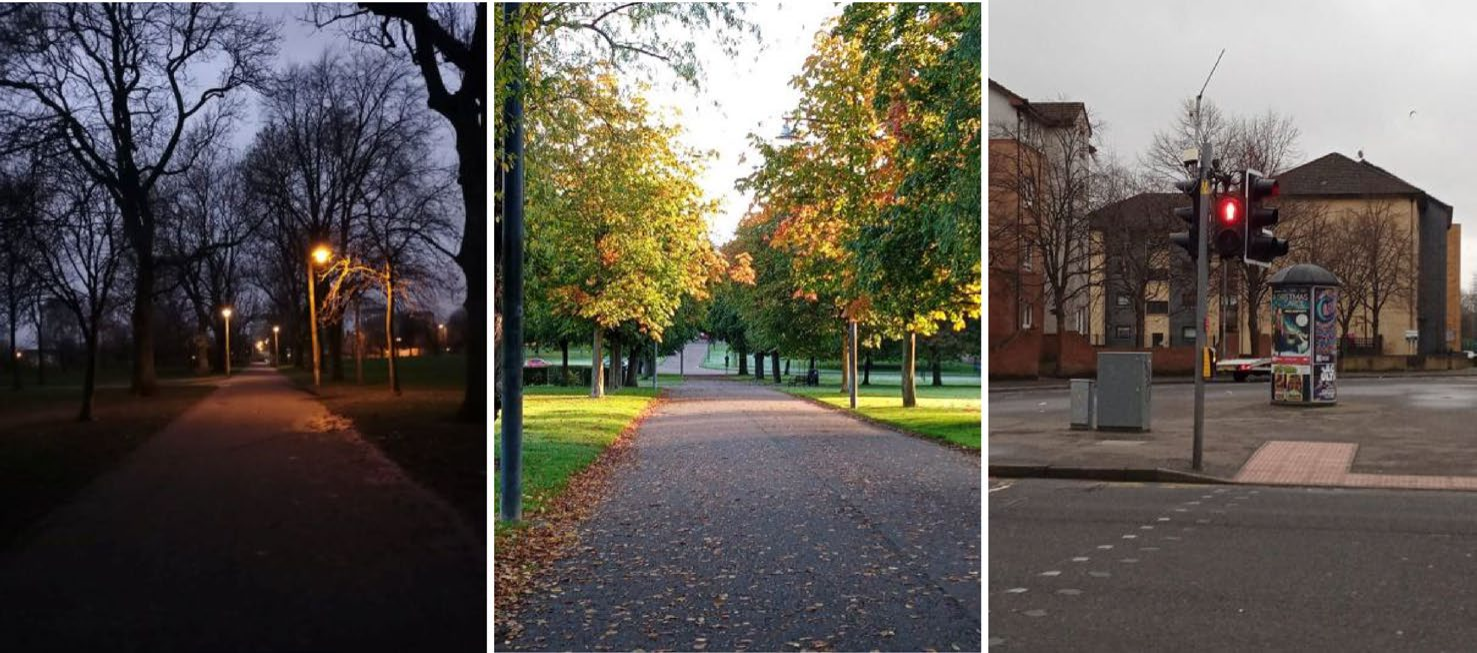
Example photographs used to facilitate discussion.

The images represented environmental influences of walking outlined in previous literature (e.g., dogs, weather, green spaces, roads, the dark); three example images are provided in Figure 1. During the interview, the researcher asked the participant to select the pictures that made them think about times they had been on a walk, or those that they thought may help with walking or make it difficult. The images were then discussed to gain insight on why the participants selected these photographs. The researcher then followed a semi‐structured interview schedule to gain insight on opportunities, motivations, and capabilities of walking.

The interviews could be conducted in person or remotely using Zoom (a video conferencing platform). The audio of the in‐person interviews was recorded with a Dictaphone, and the remote interviews were recorded over Zoom. The audio recordings allowed the researcher to transcribe the audio verbatim and enabled the transcriptions to be checked for accuracy against the initial recordings. A reflexive approach to data collection allowed for adaptations to the methods. These adaptations included the provision of more context to the questions and greater explanation for the photo elicitation activity.

#### 2.5.3 Pathway Two: Participant Directed Photo‐Elicitation Activity and Focus Group

In situ data were gathered during a walk using photograph‐based methods to support adults with intellectual disabilities to be involved in the data collection process (Overmars‐Marx et al. 2018; St. John et al. 2018). A focus group was then used to discuss the photographs to enable understanding of collective experiences (Chinn and Balota 2023). The walking route was decided in advance and was one used by the walking group. During the walk, the participants guided the researcher to take photographs that they felt were important or related to their experiences of walking. Having the researcher take the photographs reflects an approach called guided photo‐voice which was developed to reduce some of the barriers to photograph methods with adults with intellectual disabilities (Overmars‐Marx et al. 2018). A focus group took place at the organisation's venue 2 weeks after the walking activity. Images were printed for each participant and the researcher asked questions about the photographs and how they related to walking. The questions were shaped by observations made by the researcher during the walking activity.

### 2.6 Analysis

Data were analysed using the framework approach to thematic analysis as the goal was to map influences of walking onto the existing framework of the COM‐B (Gale et al. 2013). Analysis was aided through NVIVO 11 qualitative data management software. Following an initial familiarisation stage, a preliminary coding framework was developed using the first four transcripts (Gale et al. 2013). Inductive coding were initially used to understand influences of walking from the perspective of people with intellectual disabilities derived directly from the data. These codes were refined, reviewed, and then categorised under the COM‐B. The framework was then used as a guide and new influences of walking were added when discovered. Throughout this iterative process, there were frequent discussions and meetings to ensure the analysis reflected the data, and the influences were appropriately mapped onto the COM‐B.

The categorisation onto the COM‐B involved reference to the Theoretical Domains Framework (TDF). The TDF is based on the synthesis of existing theories relating to behaviours and behaviour change, and is often used alongside the COM‐B to expand the influences (Cane et al. 2012; Michie et al. 2014). For example, psychological capabilities in the COM‐B are associated with knowledge, cognitive and interpersonal skills, memory, attention and decision processes, and behavioural regulation in the TDF (Michie et al. 2014; Atkins et al. 2017). As both the COM‐B and TDF were not based on the synthesis of research including people with intellectual disabilities, these frameworks were only used as a guide to inform allocation. Categorisation onto the COM‐B was an iterative process based on discussions among the research team who have experience of research using the COM‐B and physical activity interventions for adults with intellectual disabilities.

Once the coding framework was applied to the remaining interview transcripts, a framework matrix was produced using NVIVO. The influences identified for capabilities, opportunities, and motivations to go on a walk were presented by cases (by each participant included in the study). The framework matrix helped to explore the important participant characteristics, such as gender, age, and SIMD. An example excerpt from the matrix for ‘physical opportunities’ has been provided in Appendix 2; however, full demographic information has been removed from this coding framework to help preserve confidentiality.

The focus group data were analysed using the coding framework developed for the interviews as a guide. The data was treated as separate but complementary to the data collected during the interviews. Therefore, new codes were generated when analysing this data, and not all the existing codes were relevant.

### 2.7 Inclusive Research Practices

Throughout the research process there was close collaboration with Values into Action Scotland (VIAS), with a member of VIAS attending all team meetings and involved in all decisions made. There was a separate patient and public involvement (PPI) meeting with people with lived experiences once there were preliminary findings from both the interviews and focus group. This was held in May 2023 at the lead researcher's University, with financial compensation provided for the participants' time. PPI consisted of three adults with intellectual disabilities, with one person attending to provide additional support. The researcher produced easy read slides which were approved in advance by VIAS. Feedback was given on the initial interpretation of the findings, which were integrated into the results.

## 3 Results

### 3.1 Results for Pathway One: Semi‐Structured Interviews to Identify Influences of Walking Applied to the COM‐B Model

A total of 12 adults with mild to moderate intellectual disabilities were interviewed, and nine participants had a source of support present. As can be seen in Table 2, there was a spread of ages with more men taking part than women (*n* = 5 women). Based on postcode, a majority of participants lived in the most and second most deprived areas of Glasgow, with only one participant living in an area classified as least deprived. One participant was non‐ambulatory using a wheelchair during walks. Interviews were either in‐person (*n* = 10) or over zoom (*n* = 2). The in‐person interviews were conducted in agreed safe locations, such as the University campus or the participant's home. Interviews lasted between 20 min and 1 h and 6 min.

**TABLE 2:** Demographic characteristics of interview participants.

Presence of a support during interview	
Yes	9 (75%)
If yes, paid support staff	6 (66.7%)
If yes, family caregiver	1 (11.1%)
If yes, other^a^	2 (22.2%)
Age	
Range	23–66 years
Mean	39.92 years
Standard deviation	18.18
Gender	
Women/female	5 (41.7%)
Men/male	6 (50%)
Identifies as a man	1 (8.3%)
Residential setting	
Lives on their own with support	5 (41.7%)
Lives on their own	2 (16.7%)
Lives in supported living	2 (16.7%)
Lives with parents	2 (16.7%)
Lives with partner and children	1 (8.3%)
Scottish Index of Multiple Deprivation	
1 = Most deprived	5 (41.7%)
2	5 (41.7%)
3	—
4	—
5 = Least deprived	1 (8.3%)
*Missing data* ^*b*^	*n* = 1 (8.3%)

^a^ Two participants requested to support each other.

^b^ Participant did not disclose postcode.

### 3.2 Capabilities

#### 3.2.1 Psychological Capabilities

Psychological capabilities relate to knowledge, psychological skills, and mental processes (Michie et al. 2014). The influences categorised as psychological capabilities relate to the perceived impact of limitations in adaptive skills, poor mental health, and identifying as autistic (Table 3). Participants described adaptive skills, such as conceptual skills relating to money, and social skills including gullibility and naivety, as reducing their capability to walk independently. As a result of these limitations in adaptive skills, participants disclosed feeling anxious due to previous experiences of throwing away money, and because of their perceived safety and self‐described vulnerability:

I know I'm quite vulnerable, even though I hate that word, I am classed as vulnerable. So like if a stranger come up to me and say do you want to come to my place or do you want to come see my dog …I might tend to go with a stranger if they asked me. [Participant 8]

Limitations in adaptive skills also contributed having reduced autonomy and a reliance on support: ‘Oh aye. Like I'm in Saturday and Sunday myself. By Monday I'm climbing the walls to get out of the house, do you know what I mean?’ [Participant 11] Additionally, two participants identified as autistic, which contributed to psychological capability for walking in large crowds or when walks disrupted routine. Over half of the participants discussed mental health as impacting on the psychological capabilities as low mood prevented them from leaving their home: ‘Yeah. I don't go. I won't even get ready if I don't feel great. You know, I'll just stay in my jammies and that's being honest with you.’ [Participant 4].

**TABLE 3:** Walking influences mapped under Capabilities.

Influences of walking applied to the COM‐B model			
COM‐B component	COM‐B sub‐component	Influence of walking	Description
Capabilities	Psychological capabilities	Impairments in adaptive skills associated with having intellectual disabilities (7/12)	Walking is impacted on by difficulties with abstract concepts, such as money; impairments in social skills making participants more vulnerable; and being unable to walk independently.
		Poor mental health (7/12)	Having low mood and/or anxiety results in reduced capabilities to go out on a walk.
		Self‐reported autism (2/12)	Two participants identified as being autistic, which was described as having an impact on walking through stress caused by large crowds and disruption to routine.
	Physical capabilities	Experiencing poor physical health (7/12)	Participants report experiencing poor physical health which can impact on capability to go on a walk.
		Older age impacting on walking (2/12)	Older age is associated with poorer health and mobility issues which impacts on ability to go on a walk.

*Note:* (*x*/12) indicates the proportion of the 12 participants that reported an influence.

#### 3.2.2 Physical Capabilities

Physical capabilities in the COM‐B are defined as relating to physical skills, strength, and stamina (Michie et al. 2014). During the interviews, people described the impact of poor physical health and co‐morbidities (Table 3). Reported health conditions included hypermobility which caused pain and discomfort, mobility problems, lingering discomfort after previous injuries, asthma, chronic obstructive pulmonary disease (COPD), visual impairments and health issues associated with poor physical fitness and having obesity. For example, one participant stated: ‘I'm like… ready for a walk, then I'm ready for a walk. But if I get up and [fake cough noises] then I'm no ready for a walk.’ [Participant 11] Two participants also reported that their older age influenced their physical capability as being older contributed to poorer health and mobility problems (Table 3).

### 3.3 Opportunities

#### 3.3.1 Physical Opportunities

Physical opportunities relate to the physical resources people have access to, and more conceptual resources, such as time (Michie et al. 2014; Table 4). One of the most highly reported influences was access to green spaces, such as parks. Parks were seen as giving the opportunity to be around nature and having benefits to wellbeing:

There's a wee park up beside me you know, so I walk there and then I walk back down. You know, and just kind of sit about a wee while and then say right. It's not even just that, the fresh air gives you…makes you feel good. [Participant 4]

**TABLE 4:** Walking influences mapped under Opportunities.

COM‐B component	COM‐B sub‐component	Influence of walking	Description
Opportunities	Physical opportunities	Access to green spaces (12/12)	Having access to green spaces and parks gives people with intellectual disabilities opportunities to go on a walk.
		Walking in the dark (11/12)	Although some participants felt that walking in the dark was relaxing, other participants felt unsafe.
		The importance of weather (10/12)	The weather heavily influences opportunities to go on a walk.
		Roads and poorly maintained paths (10/12)	Roads with fast cars and bad traffic can be barriers to walking, with poorly maintained paths being tripping hazards.
		Unsafe walking routes (7/12)	Some walking routes can be unsafe for walking reducing opportunities to go on a walk.
		Need access to suitable clothes and water (3/12)	During a walk, you need to have access to resources, such as suitable clothing.
		Need for wider funding and organisational support (2/12)	There needs to be more funding available to services for people with learning disabilities and greater support provided.
	Social opportunities	Having consistent social support is needed (12/12)	Having someone to provide practical social support or helping plan walks is important. However, not everyone has access to this level of support for their intellectual disabilities.
		Other people can be a barrier to walking (12/12)	People holding stigmatised perceptions of intellectual disabilities is a barrier to walking. Additionally, a general lack of awareness of others in large crowds can prevent walking.
		Dog walking can be both a facilitator or a barrier (11/12)	Having a dog, or knowing someone with a dog, gives you opportunities to go on a walk. However, dogs can make walking more challenging as they can pull and result in safety issues.
		Having access to groups and organisations provides opportunities but they are not available to all (7/12)	Walking groups and organisations for people with intellectual disabilities increase social opportunities. However, not everyone has access and some groups closed during the pandemic.

*Note:* (*x*/12) indicates the proportion of the 12 participants that reported an influence.

Walking in the dark was seen as either peaceful or unsafe because of poor visibility or due to other people. Gender differences in perceptions of walking in the dark became evident during the analysis. Although two men taking part did appraise the dark as unsafe, the other men participating reported no safety concerns or felt the dark made walking peaceful and relaxing: ‘Really good, I like going out for a walk at night‐time…because it's quiet and nobody's around’. [Participant 10, Man]. Importantly, one of the men felt that walking in the dark was unsafe, but these risks were attributed to women:

…especially if you were a lady. I've known so many ladies that sadly has walked home by themself at night through like dark allies… The dark ally or the darks path and sadly they've been sexually assaulted, or they've been… it's been all over the Glasgow live [Participant 5, Man]

Reflecting this, all of the women taking part appraised walking in the dark as unsafe with this predominantly linked to other people being dangerous: ‘It's kinda… because it is getting frightening now when you go out, if you understand. You know because you're afraid to go out at night now because you don't know what's going to happen anymore.’ [Participant 4, Woman] Women also described wider safety concerns when walking in isolated areas and on ‘lonely roads’:

I know, but it's an empty street as well remember. [Participant 6, attending support] ‘Nope. It's a one in five chance that you get attacked and have you read the reports today? There's been more attacks on Glasgow than there was like ten years ago’. [Participant 6, Woman]

While describing physical opportunities, participants also referred to tangible resources, in the form of suitable clothes and water. With participants also referencing the need for wider funding and organisational support, which related to walking groups. However, the need for tangible resources highlighted accessibility concerns, with not everyone having access to the same opportunities and previously available options closing due to the COVID‐19 pandemic: ‘Well, it's just where everything was closed due to COVID’. [Participant 6].

#### 3.3.2 Social Opportunities

Social opportunities are defined as relating to interpersonal influences, cultural and social norms (Michie et al. 2014; Table 4). Social support was identified as a central social opportunity enabling access to walking places and provision of encouragement. However, not everyone had the same opportunities or access to social support: ‘Here's the problem – I've never had support. Not in my life for anything…’ [Participant 2]

All participants reported the negative impact of other people, as participants described experiencing harassment and discrimination by members of the public and within the wider perceptions of society: ‘See soon as you get diagnosed with a learning disability you're supposed to go inside, out the road because you're no’ supposed to have the common sense or the way to speak or whatever…’ [Participant 4]

Having a dog, or knowing someone with a dog, facilitated walking; however, dogs can pull and make walking difficult. Walking was also facilitated by the presence of walking groups and organisations providing support for people with intellectual disabilities. However, these groups were not available to everyone and many groups closed during the COVID‐19 pandemic.

… it's nice to walk in a…we enjoyed that again walking in a crowd? Because you heard everybody chatting and so it was nice to chat to the other people as well. No just me. Isn't it, you enjoyed that? [[Participant 6], Attending support]

Additionally, level of deprivation contributed to variations in access to these resources. One participant lived in an area classified as ‘least deprived’. Compared to the other participants, the participant had more opportunities through access to football clubs, voluntary working positions, dance classes, youth clubs and walking clubs. Emphasising the impact of financial resources and support availability contributing to disparities in opportunities for walking.

### 3.4 Motivation

#### 3.4.1 Reflective Motivation

Reflective motivation relates to reflective processes, such as making plans (Michie et al. 2014). There were seven influences during the interviews categorised under reflective motivation (Table 5). Across the participants there were mixed intentions towards going on a walk with not everyone being self‐motivated. Perceived benefits of walking were attributed by most participants to social opportunities which enabled participants to meet new people, talk to others while walking and to help foster social connectedness: ‘That's right, it makes you happy, chatting with your friends. You look forward to it [walking group], don't you?’ [[Participant 12], Attending support]. There were also benefits relating to mental wellbeing, as walking was mindful, relaxing and a time to think: ‘Happy. It's just I feel I find it is a good copinsm [coping mechanism] to de‐stress and to relax. There you don't need to worry about anybody or anyone’ [Participant 5].

Additionally, reflective motivation was increased when there was a purpose or a goal, with the most reported being walking to the shops. Another recurrent influence was the impact of walking on physical health benefits, with walking described as being ‘good’ for you, contributing to weight loss and being a form of exercise. ‘Well… when you're sitting you've just got your belly, it's just sitting, but when you're walking you're burning the muscles in your belly and it burns all the fat away. All the fat away… I'll need to start again.’ [Participant 11]

**TABLE 5:** Walking influences mapped under Motivation.

COM‐B component	COM‐B sub‐component	Influence of walking	Description
Motivation	Reflective motivation	Mixed intentions to go on a walk (11/12)	There are mixed intentions to go on a walk, with some people highly motivation and others feeling like it is a chore.
		The perceived benefits of walking with others (11/12)	Walking gives you an opportunity to talk to others and meet people, which fosters social connectedness and helps to deal with loneliness.
		Walking helps with mental wellbeing (10/12)	Walking is a mindful and relaxing activity that can help to improve mental wellbeing.
		Goals and having a purpose for a walk (9/12)	Having a reason to go on a walk and having a specific goal can help foster motivation.
		Walking has health benefits (7/12)	Walking is good for you, it can help you loose weight and eat what you want.
		Wanting to see something new and new scenery (4/12)	Motivated to go on a walk by the opportunity to see something new.
		Not wanting to mess with routine and other priorities getting in the way (3/12)	Routine is very important and there are often other priorities that get in the way.
	Automatic motivation	Rewards and a sense of achievement foster motivation (8/12)	Rewards, primarily food, are a source of motivation for walking. Additionally, going for a long walk can give a sense of achievement.
		Fear and anxiety can impact on walking. (7/12)	Fear of falling, fear of being in busy crowds due to COVID, or fear caused by previous negative interactions with members of the public can reduce motivation to go on a walk.

*Note:* (x/12) indicates the proportion of the 12 participants that reported an influence.

#### 3.4.2 Automatic Motivation

Automatic motivation is defined as ‘automatic processes’, which includes emotional reactions (Michie et al. 2014). Two influences were categorised under automatic motivation (Table 5). The most consistent influence related to having rewards and a sense of achievement, with the main reward relating to food or a beverage as a treat to look forward to after walking: ‘Yeah, you get a coffee and cake when you get back and you chat more, don't you? You sit down, have a rest, and have a coffee and cake.’ [Participant 12, Attending support].

Seven participants also described the impact of fear and anxiety on their walking, with fear tied to bad experiences with members of the public that made them feel unsafe, anxiety over catching COVID‐19, and fear of falling and becoming injured.

So and then I get feared [scared] to go out if that happens. I mean, being honest with you, I fall in the house so I do. I'm just… I'm clumsy in the house do you know what I mean, because sometimes you trip over your own feet. [Participant 4]

### 3.5 Results for Pathway Two: Participant Directed Photo‐Elicitation Activity and Focus Group

Participants involved in this pathway were not involved in pathway two. Five participants took part during this stage which included four men and one woman, aged between 24 and 40 years old. All participants were part of a walking group facilitated by a non‐profit organisation for people with intellectual disabilities. One of the participants was non‐ambulatory and used a wheel chair. During data collection, a source of support was present with each participant along with the organiser of the walking group. Participants had difficulties completing the full demographic questionnaire, so only gender and age were recorded.

### 3.6 Capabilities

The focus group described physical capabilities that impacted on capacity to go on a walk. Participants described that some people had visual impairments and emphasised that walking routes need to be accessible, with one member of the walking group trained to identify potential hazards (Table 6, Image 1). One of the participants was also non‐ambulatory but, in the past, wheeled themselves during walks. The participant showed the researcher the broken brackets on their wheelchair (Table 6, Image 2), which were described as restricting the participant's independence.

**TABLE 6:** Example photographs and illustrative quotes.

*Image 1. Having a trained walk leader provides support and keeps people safe*	
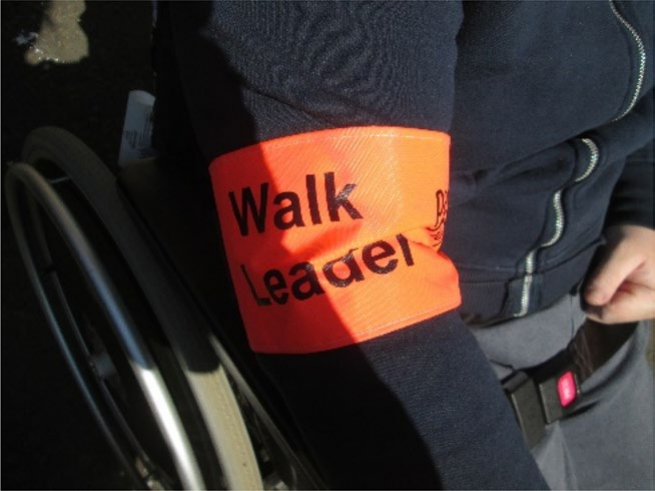	‘I would also… I would also need to know the routes, emm the safety aspects. [Focus group, Participant 1] … Keep people safe. [Focus group, Participant 4]’
*Image 2. Broken bracket on wheelchair impacting on physical capabilities*	
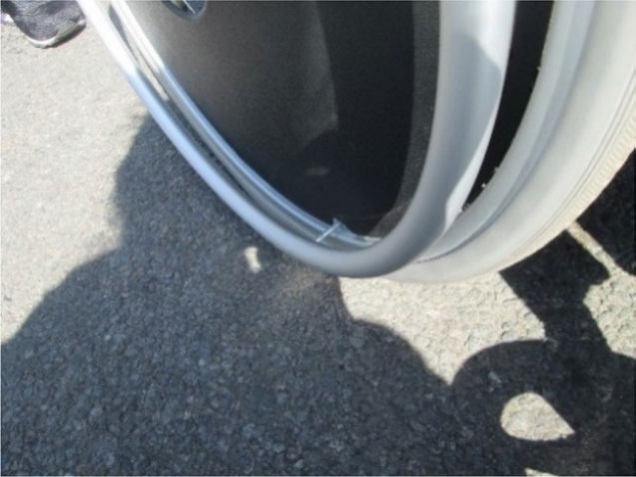	‘It was really shaky, and… It was really shaky… but that day, that was really risky.’ [Focus group, Participant 1] ‘Especially [Focus group, Participant 1], that's your mode for you being able to use your hands to wheel yourself, isn't it? And that's taking away your independence’. [Walking group organiser]
*Image 3. Parks are good places to walk when the weather is good*	
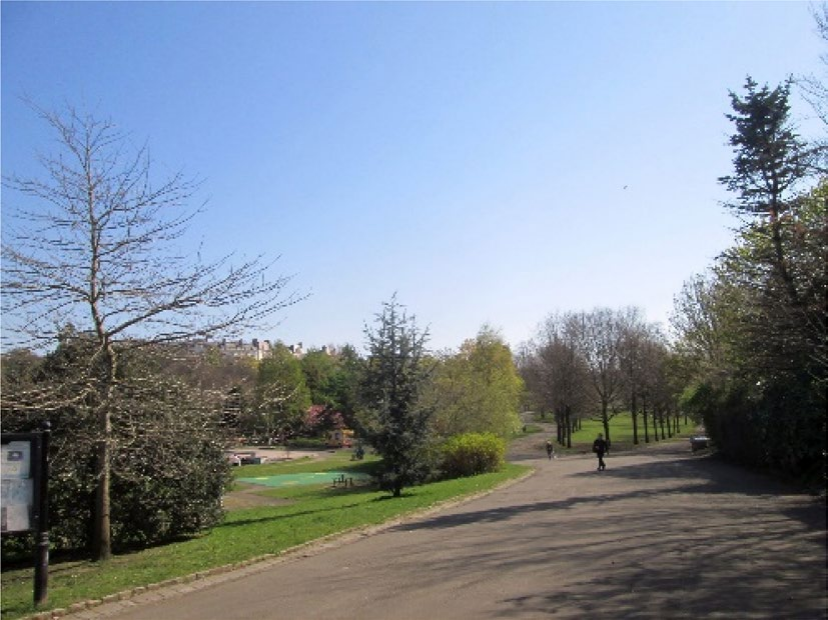	‘I liked it because em… I liked pretty much all of it really’ [Focus group, Participant 1] ‘It was good that the weather was nice as well’. [Focus group support]
*Image 4. Uneven pavement because of tree roots*	
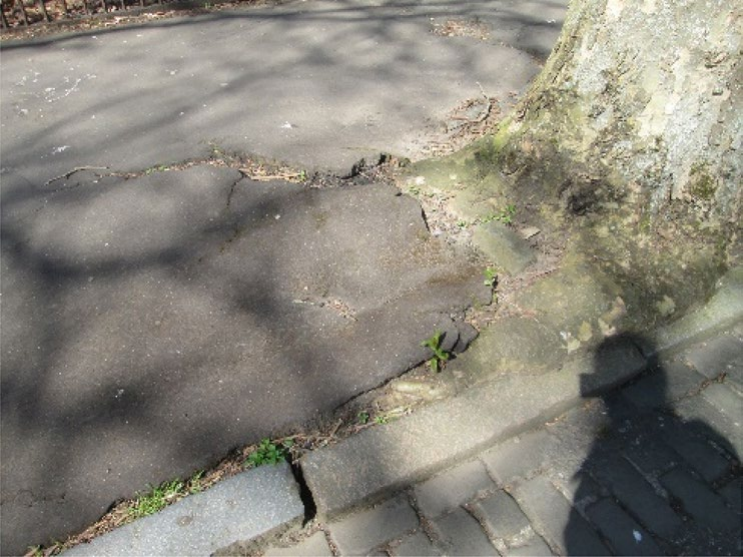	‘The roots that are coming through the concrete could trip over them, they could trip over things’. [Focus group, Participant 1]
*Image 5. Other park users can present safety issues*	
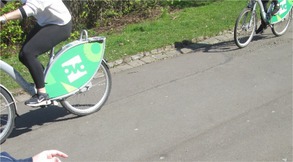	‘The fact is eh that it could be a hazard because if… if anyone em… how could I put this easily? Em… well if someone was to just go right out in front of them, or they could have toppled over and hurt themselves, or it could have em… been… it could have been anybody that could have done em… could have done damage to any one of us or themselves’. [Focus group, Participant 1]
*Image 6. Police presence associated with safety but cars in the park are a hazard*	
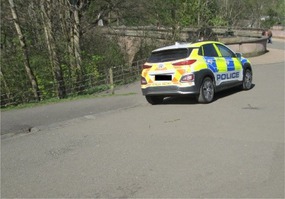	‘Well this was a hazard the police shouldn't have been parked in the middle of the pedestrian highway because there was spaces where they could have parked their car and they wouldn't have had to park in the middle of the highway’. [Focus group, Participant 1] ‘No one's… *No one* is above the law, not even… not even the… not even police officers are above the law’. [Focus group, Participant 1]
*Image 7. Flowers and nature provide motivation for walks*	
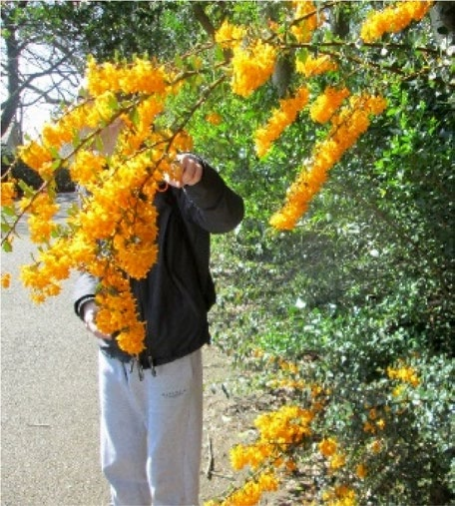	‘When you were on the walk, how did seeing flowers make you feel? [Researcher] Happy. I like gold. [Focus group, Participant 4]’
*Image 8. Flowers and nature provide motivation for walks*	
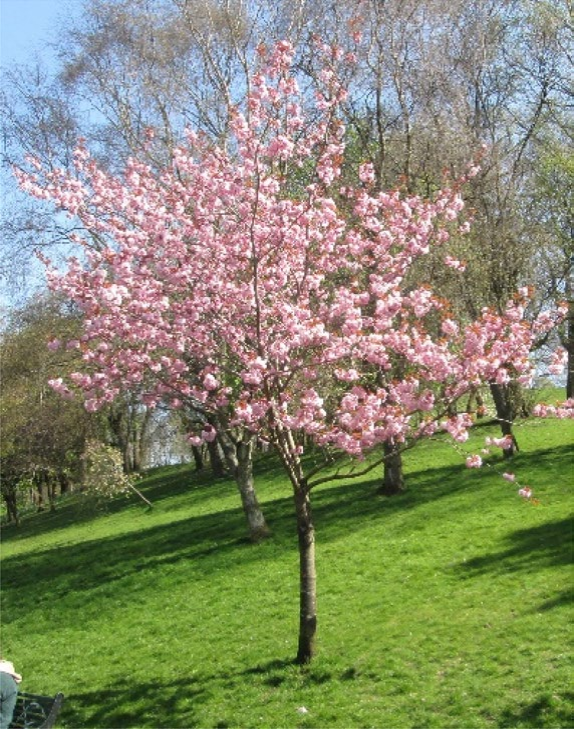	‘Happy’. [Focus group, Participant 4] ‘And why is that?’ [Researcher] ‘Because the colours… Nature’ [Focus group, Participant 4] ‘All the pink stuff and then it falls on the grass. Oh the pink stuff …the blooms’. [Focus group, Participant 2]
*Image 9. Walking as group fosters social connectedness*	
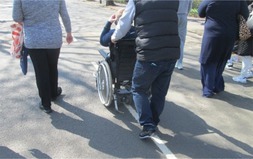	‘Being together’. [Focus group, Participant 4] ‘[Focus group, Participant 2] looks forward to it. Before it even starts, he likes talking about it before he goes’. [Focus group, support] ‘Seeing everyone’. [Focus group, Participant 5]

### 3.7 Opportunities

Parks were considered by participants to be good places to walk, and on the day of the walk, the weather was pleasant, which facilitated walking (Table 6, Image 3). During this time, potential hazards were pointed out to be taken as photographs. These were identified by the walking leader who was involved in actively risk assessing (Table 6, Image 1). The risks related to uneven pavements, cyclists in the parks, hills, the potential for trees to fall over, and crossing roads. One of the hazards included tree roots damaging the pavement and other park goers, such as cyclists (Table 6, Images 4 and 5).

#### 3.7.1 Social Opportunities

During the walk, there were police in the park and the presence of police cars was seen as a potential hazard but the police were described as helping to keep people safe (Table 6, Image 6). The organisation had facilitated one of the group members to receive formal training to become the designated walk leader (Table 6, Image 1). The walk leader took the role seriously and identified potential hazards during the walk which also helped other participants to feel safe.

### 3.8 Motivation

#### 3.8.1 Reflective Motivation

The walk took place during the spring when flowers were in bloom. The researcher was directed to take photographs of the cherry blossoms and other flowers as the sight made the participant ‘happy’ (Table 6, Images 7 and 8). For one participant, having the active role of ‘walk leader’ enforced a sense of responsibility and purpose: ‘Every time we're on a walk then what that does is then that gives me the responsibility that as a walk leader that gives me the responsibility for like everyone …’ [Focus group, Participant 1] Overall, being part of the walking group was a main source of motivation (Table 6, Image 9). It fostered social connectedness and helped provide avenues for other opportunities, such as training to actively support the group or being supported to incorporate other interests.

### 3.9 Findings Prioritised by People With Lived Experiences of Having Intellectual Disabilities

The PPI group agreed with the findings and felt like it captured their experiences of walking. They emphasised that walking in a group helps improve the walking experience, and that there should also be a reason for a walk, such as walking to the shops. The involvement group stressed that everyone has individual needs, and that it is important that everyone feels included.

## 4 Discussion

This study was the first to use the COM‐B model to develop a behavioural diagnosis of walking among adults with mild to moderate intellectual disabilities. The findings emphasise the complexity of understanding influences of walking for adults with intellectual disabilities and the need for a flexible theoretical framework. The multiple capabilities, opportunities, and motivations were interacting, with many of the influences identified as unique to the experience of having an intellectual disability.

People involved in the study described how limitations in adaptive skills impacted on their psychological capability to independently walk outdoors. Reduced autonomy, safety concerns, and a reliance on social support can ultimately restrict the opportunities people with intellectual disabilities have for walks (Brooker et al. 2015; Mitchell et al. 2018). Additionally, the psychological and physical capabilities of poor mental health, poor physical health, and mobility issues impacted on capacity for walking. These capabilities interact with the physical opportunity for accessing walking routes, with not everyone having the same access to green spaces, safe walking routes, and maintained paths. Most participants also resided in areas of Glasgow with greater deprivation, which has been linked to poorer quality of outdoor green spaces in Glasgow (Baka and Mabon 2022), and lower green space provision in urban areas of Scotland and England (Ngan et al. 2025), emphasising a need to address the inequalities in access opportunities.

Walking opportunities may also be impacted by gender, with women participants more likely to perceive walking in the dark or on ‘lonely roads’ as unsafe. In research involving people without intellectual disabilities, women and girls feel less safe walking alone in the dark and in greenspaces like parks (Office of National Statistics 2022; Barker et al. 2022). The role of gender requires further attention, as research involving people with intellectual disabilities has been criticised for being ‘gender blind’ (O'Shea and Frawley 2020; Dusseljee et al. 2011; Umb‐Carlsson and Sonnander 2005; Westrop et al. 2019; Westrop et al. 2024).

For all participants, walking was tied to the social opportunity of having consistent social support. However, receiving social support relies on the capabilities, opportunities, and motivation of support providers, along with the wider financial resources available to access meaningful support (Bossink et al. 2017, 2019; Westrop et al. 2024). Social opportunities also related to the negative impact of members of the public, as harmful interactions contributed to the automatic motivation of fear and anxiety when walking.

Nevertheless, opportunities for meeting other people were a main source of reflective motivation and identified as a key priority by the PPI group of people with lived experiences. These findings have important implications as adults with intellectual disabilities are at increased risk of experiencing loneliness and have reduced social connections (Alexandra et al. 2018; Bishop et al. 2024; Harrison et al. 2021). There is a need to support social connectedness when developing lifestyle change interventions for adults with intellectual disabilities (Westrop et al. 2024). Access to nature and fresh air was also seen as a source of reflective motivation for walking attributed to relaxation and improving mental wellbeing. Two participants involved in the study were non‐ambulatory and identified as walkers, suggesting that walking may be conceptualised as going outdoors. There is growing evidence of the importance of accessing outdoor green and blue spaces (Geary et al. 2023; Master et al. 2022; Paluch et al. 2022), with nature‐based interventions for adults with intellectual disabilities linked to improved perceived autonomy, wellbeing, and opportunities to foster social connectedness (Rotheram et al. 2017; Kaley et al. 2019).

The reflective motivation of walking with a purpose or reason for a walk was also appraised as an important finding by the PPI group. These findings taken within the wider context of behaviour change could indicate the benefit of behaviour change techniques, such as ‘goal setting’ or ‘action planning’. Goal setting and action planning have been adopted in interventions with people with intellectual disabilities (Rana et al. 2024) and appraised as suitable for people with intellectual disabilities (Willems et al. 2017).

The PPI group emphasised that everyone has individual needs and that everyone should feel included. Involvement of people with intellectual disabilities in behaviour change research ensures the interventions are accessible and reflect individual needs (Westrop et al. 2024). People with intellectual disabilities have been excluded from health‐based research, and collaboration with experts by lived experience can help support inclusion (Bishop et al. 2024).

### 4.1 Limitations

The findings were bound to the specific experiences of adults with mild to moderate intellectual disabilities living in Greater Glasgow, limiting the transferability of the findings. Further research is required to confirm the capabilities, opportunities, and motivations for walking among adults with intellectual disabilities in different contexts. Although a potential intersection between gender and having a disability was observed, the data did not allow for consideration of other intersecting identities such as racial and ethnic identity or level of intellectual disability. The focus group also only included one woman, which reflected the ratio within the walking group.

### 4.2 Strengths and Implications

The main implication and strength of this study relates to the application of the COM‐B to understand walking among adults with intellectual disabilities. Specificity is considered important when applying the COM‐B to a behaviour (Michie et al. 2014), as there may be specific influences on walking as a behaviour compared to a broader conceptualisation, such as the physical activity of adults with intellectual disabilities (Westrop et al. 2024). Supporting recent research on the physical activity of adolescents with intellectual disabilities (McDermott et al. 2022), the COM‐B was considered to be an appropriate framework for understanding the walking behaviour of adults. However, the COM‐B, and associated BCW and TDF, were not developed based on research including people with intellectual disabilities. More research is required to understand how best to allocate the influences of walking onto the COM‐B.

A major strength and implication of this study relates to the involvement of people with lived experiences in the research. Having this involvement helped to ensure the study was accessible and helped to prioritise findings important to people with intellectual disabilities. The use of qualitative methodology further facilitated meaningful involvement and ensured there was flexibility to reflect individual preferences (e.g., option of remote or in‐person interviews).

### 4.3 Recommendations for Future Research

There is a need to adopt accessible methods to facilitate understanding of walking among people with severe and profound intellectual disabilities who were excluded from this study. Researchers must consider the impact of intersecting identities on walking experiences. It is essential for researchers to involve people with lived experiences throughout the research process to ensure findings best reflect the needs and ensure that everyone feels included. More research is required in exploring the applications of the COM‐B in a behaviour change context for adults with intellectual disabilities.

## 5 Conclusions

The COM‐B is a flexible framework that can be applied to understanding walking for adults with intellectual disabilities. The findings emphasise the complexity of behaviour change in this population, due to many interacting capabilities, opportunities, and motivations. Involvement of people with lived experiences helps to identify the key findings important to people with intellectual disabilities. Future researchers should ensure that all experiences are captured by involving the people experiencing multiple intersecting sources of disadvantage.

## Conflicts of Interest

The authors declare no conflicts of interest.

## Supporting information


**Data S1:** jar70105‐sup‐0001‐Supinfo1.docx.

**Data S2:** jar70105‐sup‐0002‐Supinfo2.docx.

## Data Availability

The data that support the findings of this study are available from the corresponding author upon reasonable request.
