# Supplementary material for: Understanding Capabilities, Opportunities, and Motivations of Walking for Physical Activity Among Adults With Intellectual Disabilities: A Qualitative Theory‐Based Study
Source: J Appl Res Intellect Disabil. 2025 Jul 30;38(4):e70105. doi: 10.1111/jar.70105 (PMC12309153; doi:10.1111/jar.70105)
Supplement: Supplementary file 1 — Data S1: jar70105‐sup‐0001‐Supinfo1.docx. [file JAR-38-e70105-s001.docx]

Supplementary file 1. **Interview schedule format**

**Note: prompts, follow-up questions and probes used. Rephrase questions if there is difficulty understanding.**

Purpose: understand your experiences of waling for physical activity. To understand the opportunities you have, what motivates you, and what makes it more easy or difficult to go on a walk.

Walking: decide to go for a walk for leisure / choosing for a walk instead of going by car etc.

Discussion about photographs

“These pictures are here to help us think about your own experiences of walking. There are no right or wrong answers and some pictures may not be important to you. Do any of these pictures make you think about times that you have been on walk”
One photograph presented and discussed at a time.

- Why did you choose / take this picture?
- What does this picture make you think about?
- What does this picture tell us about your experiences of walking?

Behavioural diagnosis questions

| **Opportunities to go for a walk**, if there are any things in the environment and community that may help or stop walking, or if other people impact on your walking. | How easy is it for you to go for a walk?  *What makes it easy? What could make it easier?*  How difficult is it for you to go on a walk?  *What makes / could make it difficult?*  How much social support do you get to go on a walk?  How could people help you go for a walk? |
| --- | --- |
| **Motivations to go for a walk**, such as making plans to go for a walk or believing that walking is good or bad. | Do you see yourself as someone who walks a lot?  Do you make plans to go on walks?  Does anyone else make plans to go on walks with you?  When do you go on walks?  Do you think walking is good or bad for you?  How does walking make you feel?  Do you enjoy walking?  What would make going for a walk more enjoyable? |
| **Capabilities to go for a walk**, such as physical skills and strength, along with psychological factors / mental factors and knowledge. | How do you feel physically when you go for a walk?  *How else does walking make you feel (e.g., happy / sad)?*  If you want to go for a walk, is it easy to go for one?  Where do you think are good places to go for a walk?  What skills do you need to go for a walk?  Are there any things that stop you from going for a walk?  Is there anything that could help you go for a walk? |

Debrief at the end of the interview – summarising what questions were asked. Opportunity for participants to add more information and for them to ask the researcher question
