# Supplementary material for: Understanding Capabilities, Opportunities, and Motivations of Walking for Physical Activity Among Adults With Intellectual Disabilities: A Qualitative Theory‐Based Study
Source: J Appl Res Intellect Disabil. 2025 Jul 30;38(4):e70105. doi: 10.1111/jar.70105 (PMC12309153; doi:10.1111/jar.70105)
Supplement: Supplementary file 2 — Data S2: jar70105‐sup‐0002‐Supinfo2.docx. [file JAR-38-e70105-s002.docx]

| Supplementary file 2. Example of framework matrix for physical opportunities  To preserve anonymity, only gender has been included in this table. | | | | | | | |
| --- | --- | --- | --- | --- | --- | --- | --- |
|  | A : Green spaces, such as parks, are important as having access to nature and wildlife makes walking more enjoyable | B : Need for wider funding and organisational support | C : Need to be prepared with clothes and water | D : Perceptions of walking in the dark | E : Roads and pavements can be barriers to walking as they can be unsafe or poorly maintained | F : Unsafe walking options | G : Weather determines whether it is possible to go out on a walk |
| 1 : Participant 1   Gender = Man | Likes to walk in parks and on local walking paths. |  | You can go out for walks in bad weather if you have appropriate clothing. Having water also gives you more energy. | Does not mind walking in the dark as it can be quiet and relaxing. |  | There can be dangerous walking routes that are not accessible. | Good weather helps with walking. When the ground is slippery you can fall, and the rain can make walking stressful. |
| 2 : Participant 2   Gender = Man | Likes to walk in parks because of the trees and because people are more friendly. It is relaxing. |  |  | Not bothered by walking in the dark and will go to the shops at night. | Cars do not always pay attention to traffic lights, and it can be unsafe. | In rough neighbourhoods you need to know how to "conduct yourself" | Does not want to go out when it is raining or cold, and prefers to go out when the weather is good. When the weather is cold this can cause underlying health problems to be exacerbated. |
| 3 : Participant 3   Gender = Man | Goes for a "wee dodder" in the park and to get some fresh air. |  | You need to have appropriate shoes otherwise your feet will get sore. | You have to be careful when walking at night. It is a "bit risky". | Pavements can be tripping hazards. Cars go by too fast and cross at the lights is "murder". | There can be undesirables in parks. | Being out in extreme weather, i.e., strong winds or very hot, is risky. If the weather is nice and not raining, they will go on a walk. |
| 4 : Participant 11   Gender = Man | Likes to go for walks in green spaces, such as parks and countryside walks, and likes being able to see wildlife, such as deer. |  |  |  | Paths need to be more accessible by having more benches for people to rest and take breaks. There should also be more bins. Pavements are also poorly maintained and can be tripping hazards, especially when you have visual impairments. | You need to have you wits about you when on a walk, especially in the city centre. | Would not walk in the rain, and prefers good weather (i.e., when it is sunny). |
| 5 : Participant 5   Gender = Man | Goes for walks in parks as you can be around nature. | There is a high turnover of staff because of budgeting. There needs to be more funding and support for paid support staff. They do not get enough credit. | Having a jacket is needed for walking in the cold. | Walking at night is unsafe and can be particularly dangerous for women. Women have been sexually assaulted in "the dark ally or the dark paths". | Traffic can be "very manic" and stressful. Cars do not always pay attention to pedestrians. |  | When the weather is nice, it is relaxing. Snow and ice is dangerous as it is slippery and can be risky to walk in. |
| 6 : Participant 10   Gender = Man | Walking in the park means you are around tress, see different colours and can be around dogs. |  |  | Participant likes to walk at night as it is quiet and nobody is around. | The roads can be very busy and this can make walking difficult. |  |  |
| 7 : Participant 4   Gender = Woman | Being in a park gives you fresh air and you can meet people in the park. |  |  | At night or in the dark it is dangerous to be out on a walk. Anything could happen. There need to be more lights. |  | "lonely" roads and paths can be dangerous as you do not know who is there. | Will not go out in the snow, rain or wind as they are worried about falling or being "blown down". If it is too hot they feel sick so avoid going outside. They do like walking in the summer overall. |
| 8 : Participant 7   Gender = Woman | Being around nature, seeing scenery and flowers. Likes to be in green spaces. |  |  | The night is "violent" and it is not safe to be out at night. | The pavements are unmaintained and difficult to use. This participant has a wheelchair, and described falling from their wheelchair as a result of the pavements. |  |  |
| 9 : Participant 6   Gender = Woman | Having access to parks and walking paths helps. You can see trees and birds while in green spaces. | There are less opportunities for physical activity as facilities have needed to close as they are expensive to run. |  | Participant would not go out at night as it is unsafe. As soon as it gets dark the participant is at home. The support confirms that the participant would not go out in the dark on their own. | The roads need to be fixed as there are always incidents. Cyclists can also cause problems when walking. | "Empty" streets are dangerous and there are reports of people being attacked in Glasgow. | The rain exacerbates underlying health problems, but will go out in the cold (as long as it not raining). Will walk in most weather as long as it is dry. |
| 10 : Participant 9   Gender = Woman | Parks have a nice atmosphere as you can be around dogs and listen to birds. |  |  | Participant would have difficulty walking in the dark as they problems with their vision. | Cars and buses can knock you over, and crossing the road can be dangerous. Pavements can also be tripping hazards. |  | When the weather is sunny it is nicer to go out for walks. If you go for a walk in the ice you can fall and in the rain you want to stay inside. |
| 11 : Participant 12   Gender = Woman | Frequently goes for walks in the park. Parks are green spaces and the trees / wildlife make walking better. Additionally, the parks are quieter and better places to go. However, good parks require the car. |  |  | The participant describes not being bothered by walking in the dark. However, the support present (participants mother) emphasises that she cannot go out in the dark alone. The participant feels safer as she has a light with her (harry potter wand). | Crossing at the road can be unsafe and you need to look both ways. | Isolated walking routes should be avoided as they do not feel safe. | Will not go for a walk in the rain voluntarily. Enjoys going for walks when it is sunny. |
| 12 : Participant 8   Gender = Identifies as a man | Being around nature is good for you. You can listen to birds and see new things. |  |  | Participant would feel uncomfortable being out in the dark as "people get hurt or abducted at night". | Need to be careful when crossing a road. |  | Walking in the ice is dangerous as it is slippery, and would not want to go in a walk in the snow or the rain. |

Notes: Colour green = facilitator; colour orange = barrier; colour blue = neutral; colour purple = mixed findings
